# Using the Updated EWGSOP2 Definition in Diagnosing Sarcopenia in Spanish Older Adults: Clinical Approach

**DOI:** 10.3390/jcm10051018

**Published:** 2021-03-02

**Authors:** Anna Arnal-Gómez, Maria A. Cebrià i Iranzo, Jose M. Tomas, Maria A. Tortosa-Chuliá, Mercè Balasch-Bernat, Trinidad Sentandreu-Mañó, Silvia Forcano, Natalia Cezón-Serrano

**Affiliations:** 1 Department of Physiotherapy, University of Valencia, 46010 Valencia, Spain; anna.arnal@uv.es (A.A.-G.); merce.balasch@uv.es (M.B.-B.); trinidad.sentandreu@uv.es (T.S.-M.); natalia.cezon@uv.es (N.C.-S.); 2 Research Unit in Clinical Biomechanics (UBIC), University of Valencia, 46010 Valencia, Spain; 3 Hospital Universitari i Politècnic La Fe, 46026 Valencia, Spain; sforcanosanjuan@gmail.com; 4 Physiotherapy in Motion, MultiSpeciality Research Group (PTinMOTION), University of Valencia, 46010 Valencia, Spain; 5 Department of Methodology for the Behavioral Sciences, University of Valencia, 46010 Valencia, Spain; Jose.M.Tomas@uv.es; 6 Advanced Research Methods Applied to Quality of Life Promotion (ARMAQoL), University of Valencia, 46010 Valencia, Spain; 7 Department of Applied Economics, University of Valencia, 46022 Valencia, Spain; angeles.tortosa@uv.es; 8 Psychological Development, Health and Society (PSDEHESO), University of Valencia, 46022 Valencia, Spain

**Keywords:** sarcopenia, older adults, diagnostic criteria, clinical

## Abstract

Recently the European Working Group on Sarcopenia in Older People (EWGSOP2) has updated diagnostic criteria for sarcopenia, which consist of one or more measures of muscle strength, muscle mass, and physical performance, plus an initial screening test called SARC-F. The main objective was to compare the number of cases of sarcopenia, using the different measurements and screening options. A cross-sectional study was conducted on Spanish older adults (*n* = 272, 72% women). Combining the different measures proposed by the steps described in the EWGSOP2 algorithm, 12 options were obtained (A–L). These options were studied in each of the three models: (1) using SARC-F as initial screening; (2) not using SARC-F; and (3) using SARC-CalF instead of SARC-F. A χ^2^ independence test was statistically significant (χ^2^(6) = 88.41, *p* < 0.001), and the association between the algorithm used and the classification of sarcopenia was moderate (Cramer’s V = 0.226). We conclude that the different EWGSOP2 measurement options imply case-finding differences in the studied population. Moreover, when applying the SARC-F, the number of people classified as sarcopenic decreases. Finally, when SARC-CalF is used as screening, case finding of sarcopenic people decreases. Thus, clinical settings should consider these outcomes, since these steps can make preventive and therapeutic interventions on sarcopenia vary widely.

## 1. Introduction

The prevalence and impact of sarcopenia increase with age, and consequently, global aging of the population has turned sarcopenia into a public health concern of great priority both for clinicians and researchers [1]. Thus, the concept of sarcopenia has evolved in recent years at the same time that the number of scientific publications has increased in order to identify its possible causes and consequences [2,3,4].

Although there are different international teams which have published their guidelines or consensus for sarcopenia [5], the European Working Group on Sarcopenia in Older People of 2010 (EWGSOP) guideline has been one of the most widely used and has catalyzed research activity of sarcopenia worldwide [6,7,8]. In 2018, the Working Group updated the original definition (EWGSOP2), which since then considers low muscle strength as an essential characteristic of sarcopenia, uses detection of low muscle quantity or quality to confirm its diagnosis, and regards poor physical performance as confirmation of severe sarcopenia [7].

Therefore, in this recent definition (EWGSOP2), muscle strength is brought to the forefront of the diagnostic algorithm [9]. To measure muscle strength, handgrip strength or chair stand are recommended; for measuring muscle mass, two different options are given in order to adjust Appendicular Skeletal Muscle Mass (ASM) either by height squared, weight, or body mass index (BMI); finally, for physical performance, four assessment options are given: gait speed, the Short Physical Performance Battery (SPPB), the Timed-Up and Go test (TUG), and the 400 m walk [7]. Therefore, one or more measures of muscle strength, muscle mass, and/or physical performance together with gender-specific cut-off points for some of these measurements are needed for diagnosing sarcopenia [10,11]. From the clinical perspective, it has to be taken into account that these different options imply that the correct implementation of sarcopenia diagnosis in daily clinical practices requires many factors such as acquisition and financial costs of diagnostic measurement equipment, evaluator training and knowledge, and time constraints of diagnostic measures, among other factors [12]. The assessment in sarcopenia has become a challenge for healthcare professionals in order to identify those who may benefit from intervention [13], leading to a small percentage using diagnostic measures in clinical practice [12]. Therefore, while all different options of the definition are convenient and reliable [12,14], the impact of the different measurements on case finding of sarcopenia is to be elucidated and could help transfer the diagnosis of sarcopenia from research to the clinical context [12,14].

In addition, to facilitate the detection of sarcopenia, a screening test called SARC-F has been proposed to be carried out, before performing the measurements of strength and muscle mass, as indicative of the risk of sarcopenia [15]. SARC-F consists of five questions answered by the patients themselves, so it is a simple, practical, and easily applied screening tool for older adults and for the applicant. However, the use of SARC-F is not mandatory for healthcare professionals, except with screening purposes in high-risk patients [16]. Thus, EWGSOP2 recommends the SARC-F questionnaire as a way to obtain self-reports from patients with signs of sarcopenia and as a formal approach [16].

Moreover, although in previous studies conducted in community-dwelling older adults, SARC-F has shown very good specificity to diagnose sarcopenia, its sensitivity is low, which may be not desirable for a questionnaire aimed at screening purposes [17,18,19,20]. With the intention to solve this, SARC-CalF, which adds calf circumference (CC) to SARC-F, has been suggested as an option that may significantly increase the sensitivity of SARC-F [21]. If not only community-dwelling people are studied, but also institutionalized older adults are included, a broader population is characterized and therefore clinicians have more information about the use of these tools. Therefore, they should be validated in different populations and living settings [21], plus the new EWGSOP2 definition has to be taken into account.

It was hypothesized that although there are different measurement options for each step of the algorithm of the EWGSOP2 definition, no difference in case finding will be found in older adults, allowing healthcare professionals to use the most feasible in their daily clinical practice. We also hypothesized that by not using the SARC-F, case finding of sarcopenia could be increased. Moreover, it may be increased when using the SARC-CalF instead of the SARC-F in these populations.

Therefore, the aim of this study was to compare the number of cases of sarcopenia in older adults using the different measurement options of each step of the algorithm of the European Working Group on Sarcopenia in Older People 2018 (EWGSOP2). We also aimed to evaluate the impact of using SARC-F, SARC-CalF, or no screening on the case finding of sarcopenia in Spanish older adults living in the province of Valencia.

## 2. Experimental Section

### 2.1. Study Design

A multicenter cross-sectional study was carried out between January 2019 and February 2020 in institutionalized and community-dwelling older adults, living in the province of Valencia (Spain). This study was approved by the Ethics Committee for Human Research of the University of Valencia (H1542733812827) and was conducted in accordance with the Declaration of Helsinki. This research was registered in the ClinicalTrials.gov database (ID: NCT03832608). Before entering in the study, participants signed a written consent, briefed beforehand.

### 2.2. Participants

The sample included 272 adults aged 65 or older, living in the community (*n* = 139) or institutionalized in residential facilities (*n* = 133). Candidates were not included if they: (1) had edema which could interfere with the bioimpedance analysis (BIA); (2) had a cognitive impairment measured with the Mini-Mental State Examination (MMSE) < 18 points [22]; (3) were suffering from any acute or unstable chronic disease, or had a hospital admission in the last month.

### 2.3. Sarcopenia Definition

The algorithm of the EWGSOP2 was followed for case finding and diagnosing sarcopenia and determining its severity [7]. It included the SARC-F and the measurements of muscle strength, muscle quantity, and physical performance.

The SARC-F questionnaire is composed of five items questioning strength, assistance in walking, rise from a chair, stair climbing, and falls. It is scored between 0 and 2, and it allows identifying cases with a score of ≥4 points from a total of 12 points [15].

Muscle strength was measured by:

- Handgrip strength technique, with a Jamar Plus+ digital hand dynamometer (Patterson Medical, Sammons Preston, Bolingbrook, IL, USA) [23]. Cut-off points were gender-specific for low grip strength: <27 kg for men and <16 kg for women [24].
- Chair stand, in which participants had to stand up five times as quickly as possible from a chair without stopping, with arms folded across the chest. Time (in seconds) was used for the present analyses. The cut-off point for strength was >15 s for five rises for both men and women [25].

Muscle quantity as Appendicular Skeletal Muscle Mass (ASM) was measured with BIA using the Bodystat^®^ 1500MDD (Bodystat Ltd., Douglas, UK). This device was calibrated previous to the measurements. Prior to the assessment, the following criteria were checked [26,27]: participants could not have done previous physical exercise; 2–3 h of fasting was needed, including alcohol or a large amount of water, and emptying their bladder; every metal piece was taken off; and the test was not implemented if they were wearing a pacemaker and/or had edema (diagnosed by the physician). When applying the BIA test (alternating sinusoidal electric current of 200 µA at 50 kHz), the patient was asked to lie in supine position, on a nonconductive surface, with no contact between the limbs. Electrodes were applied with an ipsilateral tetrapolar method, on previously cleaned skin. The electrodes of the upper limb were placed at the knuckles and wrist, and those of the lower limb were placed at the metatarsal head bones line and the anterior side of the ankle. ASM was calculated following Sergi’s BIA equation: ASM (kg) = −3.964 + (0.227 × RI) + (0.095 × weight) + (1.384 × sex) + (0.064 × Xc) [28]. The proposed ASM cut-offs were:

- ASM: low muscle mass was <20 kg for men and <15 kg for women [29].
- ASM Index (ASMI, defined as ASM/height squared): low muscle mass was <7.0 kg/m^2^ for men and <5.5 kg/m^2^ for women [7,8].

Physical performance of participants was measured by:

- Gait speed (m/s): participants were asked to walk along a 4 m corridor at usual speed and, if needed, using an aid [30], with <0.8 m/s being the cut-off for men and women [31,32].
- Short Physical Performance Battery (SPPB): this test assessed balance, gait, strength, and endurance. Participants were asked to stand with the feet together, semi-tandem, and tandem positions, the time they needed to walk 4 m was measured, and also the time to rise five times from sitting position [33], with ≤8 points being the cut-off for men and women [34].
- Timed-Up and Go test (TUG): participants were asked to rise from sitting position, walk a 3 m distance, turn around, walk back, and sit down, with ≥20 s being the cut-off for men and women [35].

Following these assessments, participants were classified according to the EWGSOP2 algorithm [7,8]: (1) they had probable sarcopenia with a score of ≥4 points SARC-F and low muscle strength (grip strength < 27 kg for men and <16 kg for women; or chair stand > 15 s); (2) they had confirmed sarcopenia when low quantity muscle was also detected (ASM < 20 kg for men and <15 kg for women; or ASMI <7.0 kg/m^2^ for men and <5.5 kg/m^2^ for women); and (3) they had severe sarcopenia, when low physical performance was added (gait speed < 0.8 m/s; SPPB ≤ 8 points; or TUG ≥ 20 s).

### 2.4. Additional Measurements

Anthropometric variables: Age and gender were registered; body weight (kg) was measured using a Tanita BC 601 (TANITA Ltd., Amsterdam, The Netherlands); height (cm) was assessed with a stadiometer SECA 213 (Seca Ltd., Hamburg, Germany); and finally, BMI (kg/m^2^) was calculated.

SARC-CALF consists of the same five items as SARC-F which are scored the same [36] and adds the CC that was measured as the widest circumference of calf. The CC item is scored as 0 points when the participant had more than 31 cm circumference and as 10 points if it was less than or equal to 31 cm. A SARC-CalF ≥ 11 indicates positive screening for sarcopenia [37,38,39].

All the assessments were done on the same day for each participant, and different physiotherapists took these measurements for all the samples. Intraclass Correlation Coefficients (ICCs) were calculated to know the interrater reliability, and they ranged from 0.802 to 0.985, which may be considered very good reliability (values between 0.75 and 0.90 indicate good reliability; values over 0.90 show excellent reliability) [40].

### 2.5. Applied Models

Three models were applied: Model 1: using SARC-F as initial screening; Model 2: not using any initial screening; and Model 3: using SARC-CalF as initial screening instead of SARC-F. By combining the different measures proposed by the steps described in the EWGSOP2 algorithm (Find–Assess–Confirm–Severity), 12 options were obtained (A to L), and to each one of the three models, each of their twelve options was tested (Table 1).

### 2.6. Statistical Analyses

With descriptive purposes, means, standard deviations, and 95% confidence intervals (CI) for all variables were calculated. All statistical analyses were performed with R [41], also employing the packages vcd [42] and DescTools [43]. Descriptive statistics (proportions) of multinomial variables were performed [44] including 95% CI for the proportions of each category by the method of Glaz and Sison [45,46]. Chi-square tests of goodness-of-fit and independence were also performed together with their association measures (Pearson residuals and Cramer’s V). The CI for V coefficient was bias-corrected [47]. Whenever multiple statistical tests were made, the Sidak correction was employed.

## 3. Results

### 3.1. Sample Characteristics

A total of 272 participants were included in this study. The age range for all the participants was 65–97 years, the mean age was 77.0 (8.7) years old, and according to setting, the mean was 72.3 and 81.9 years old for community dwelling and institutionalized participants, respectively. Seventy-two percent of participants (*n* = 197) were women (Table 2).

### 3.2. Analysis of the Models

**Figure 1:**
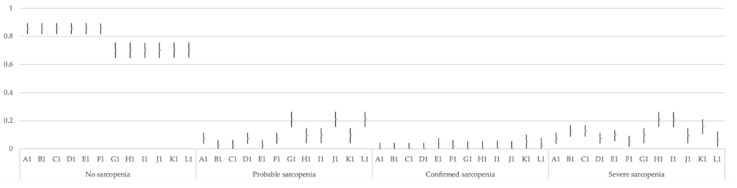
Multinomial 95% confidence intervals for the proportion of each category in the 12 options of Model 1.

**Figure 2:**
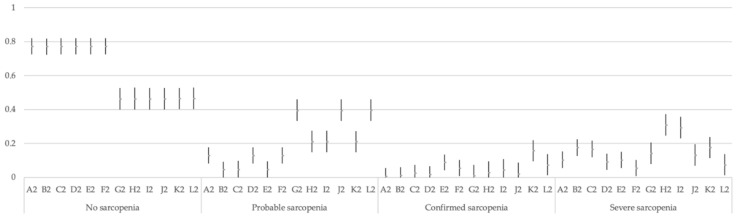
Multinomial 95% confidence intervals for the proportion of each category in the 12 options of Model 2.

**Figure 3:**
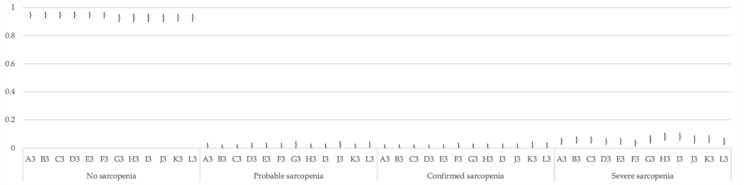
Multinomial 95% confidence intervals for the proportion of each category in the 12 options of Model 3.

For each of the three models, and each of their 12 options (A to L), 95% CI and each category of classification (no sarcopenia, probable sarcopenia, confirmed sarcopenia, and severe sarcopenia) were calculated. These CIs are presented in Figure 1, Figure 2 and Figure 3.

Once these CIs were calculated, they were averaged for each model, and each of the 12 options (A to L) in each model was compared with a goodness-of-fit chi-square test with expected probabilities the average probabilities of each model. Therefore, the 12 tests within each model (algorithm) tested whether the classification of the different steps was statistically equal or different. Table 3 offers the results of all these chi-square tests. Regarding Model 1, all but two tests showed statistical significance, indicating that the different steps of the algorithm significantly affect the classification. Model 2 tests showed significant results in all cases, and therefore this supports that the different steps of the algorithm lead to different classifications. However, in Model 3, the results showed no statistical significance, and therefore for this algorithm, the different steps do not lead to significantly different classifications.

A chi-square independence test was performed to compare the classifications into the different groups the three algorithms made. This chi-square was statistically significant (χ^2^ (6) = 88.41, *p* < 0.001), and the association between the algorithm used and the classification of sarcopenia was moderate (Cramer’s V = 0.226, 95% CI [0.177, 0.276]).

**Figure 4:**
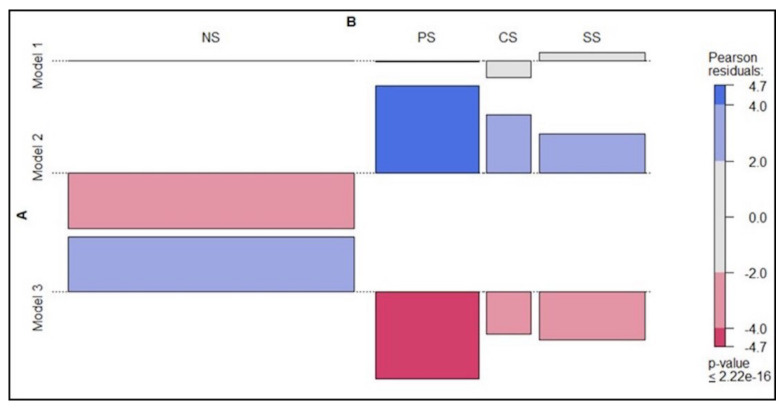
Association between the three models and the severity levels of sarcopenia (no sarcopenia (NS), probable sarcopenia (PS), confirmed sarcopenia (CS), and severe sarcopenia (SS)). Note: blue indicates positive association of row and column and red negative association.

In addition, we have analyzed how each model on the whole is associated with severity levels. Figure 4 graphically presents the association based on the Pearson’s residuals. It can be seen that Model 1 is not significantly associated with any classification as represented by the grey color. However, Model 2 is associated with the classification into the different groups of sarcopenia with a positive association (blue color) with the levels of severity, being higher with probable sarcopenia, and with negative association (red color) with nonsarcopenic older adults. On the contrary, Model 3 is associated positively (blue color) with no sarcopenia.

## 4. Discussion

The present study showed that using the different measurement options for each step of the EWGSOP2 implied differences in case finding in the studied population. These differences have been analyzed in relation to each of the steps, describing which measurements detect more or less cases of sarcopenia. Moreover, our results indicate that when applying the SARC-F, case finding of sarcopenia decreases, thus by not applying it, more cases are found, especially among those with probable sarcopenia. Finally, when SARC-CalF is used as screening, the number of people classified as sarcopenic decreases.

To the best of our knowledge, this is the first study to analyze the different measurement options of the EWGSOP2 in Spanish older adults, and the fact that there are differences in case finding has important clinical consequences. Taking into account that sarcopenia is frequently not noticeable in earlier stages [48], detecting probable sarcopenia is of paramount importance in order to be able to start intervention. In Model 1, using SARC-F, and Model 2, using no screening, there are significant differences in case finding among most of the options. When analyzing these differences, it can globally be seen that those which use the chair stand for measuring muscle strength (G to L) are the ones that find more probable sarcopenic participants. This is interesting considering that previous research has highlighted that handgrip strength seems to be used widely for the measurement of muscle strength [13]. However, it requires the use of a calibrated handheld dynamometer under well-defined test conditions [23]. Therefore, commercial dynamometers are usually limited in clinical settings by the need to purchase specialized equipment, the relative expense, and the lack of trained staff [13]. In addition, sometimes measurement of grip is not possible due to hand disability, such as with patients who are suffering from advanced arthritis or stroke [7]. On the whole, these facts could explain why only a small percentage of healthcare professionals use diagnostic measures in clinical practice as stated before [12]. On the other hand, in previous research, the chair stand has been shown to be able to provide a valid tool for assessing lower body strength [49]. This is in line with our results, which seem to show chair stand can be a reliable method for case finding of probable sarcopenia in the studied population. From the clinical approach, detection of cases as early as possible is important considering that it is better to prevent the skeletal muscle mass depletion and loss of strength and function rather than trying to restore them when they have progressed [50]. Therefore, for clinical settings where a handgrip dynamometer is not always available, the chair stand could be used as an alternative assessment of muscle strength [13]. This way, preventive strategies together with treatment interventions could be implemented before the muscle deterioration occurs [50].

After detecting probable sarcopenia cases, the second step of the EWGSOP2 algorithm evaluates muscle quantity. The EWGSOP2 consensus presents cut-off points for both ASMI (kg/height squared) [51] and ASM (kg) [29] for use when calculating muscle mass. In relation to Model 1, when analyzing the different options, there are more cases of severe sarcopenia in those options that previously have confirmed it by using the ASM (kg) cut-off for muscle quantity (B1, C1, E1, H1, I1, K1). Considering that low muscle mass is highly related to disability and frailty in older adults [52], measuring muscle mass in a precise way is crucial for confirming sarcopenia in this population. There is an ongoing debate about the preferred adjustment for muscle mass indices and whether the same method can be used for all populations [7]. For our population, the results show the ASMI is a more restrictive cut-off, whereas with the ASM, more sarcopenic participants are detected and, consequently, more are classified as suffering severe sarcopenia. This could also explain why G1, J1, and L1 show more cases of probable sarcopenia, since they are using the ASMI and therefore more participants are not being confirmed with sarcopenia and stay as probable. Therefore, some participants could present low strength, however, their amount of muscle mass would still be within the EWGSOP2 criteria, preventing categorization in more advanced stages of the pathology, as similarly stated in previous research [53]. Although the most accurate way to define muscle mass remains uncertain [54], our results show ASMI is more restrictive for our population, classifying them mostly as probable, whereas they could have been classified as severe if the ASM had been used. Thus, methods used to define low lean mass can make preventive and therapeutic interventions on sarcopenia vary widely.

In relation to Model 2, with no initial screening, there are significant differences in case finding among all of the options. When analyzing them individually, again the same trend can be found in relation to muscle strength and muscle mass, that is, chair stand detects more probable sarcopenia (options G to L) and ASMI is more restrictive (A, D, F, G, J, L). In relation to physical performance, the options which confirm sarcopenia with the ASM and then classify its severity with the SPPB or gait speed (B, C, and G to J) are the ones which detect more cases of severe sarcopenia. Detection of low physical performance predicts adverse outcomes [7], so it becomes of paramount importance for the clinical approach. However, in older populations, physical performance is frequently difficult to measure due to acute illness or because of dementia, gait disorder, or a balance disorder [55,56,57], thus finding a safe and valid assessment becomes necessary. Gait speed is considered a quick and reliable test for sarcopenia, which is why it is widely used in practice [58]. Although the SPPB also predicts outcomes [34], it is a more time-consuming test to apply and therefore, it is more used in research than in clinical practice. Therefore, and considering our results, clinicians can rely on SPPB and gait speed to detect the severe cases, although the latter can be considered as a more approachable measurement in the clinical context.

Regarding the use or not of SARC-F, it was not implemented in Model 2, and more cases were found as probable, confirmed, or severe sarcopenia. Therefore, when using SARC-F for screening in our population, it is at the expense of missing cases who would have been at least in the category of probable sarcopenia, since more cases were detected in Model 2. Although the SARC-F has shown excellent specificity [15,17,18,59,60,61], it has shown some problems in relation to its low sensitivity [17,59], which means that there is a high risk of missed diagnosis of individuals who have sarcopenia. Moreover, as noted in the EWGSOP2 definition, in clinical settings, case finding should start when a patient has symptoms or signs of sarcopenia, and in these situations, further testing is recommended, the use of any screening tool not being mandatory [16]. This is in line with our results, which suggest SARC-F does not always detect possible cases of sarcopenia in our sample.

In relation to Model 3, which screens using the SARC-CalF, our results show case finding of sarcopenic people is not increased. Moreover, it is the model with which a lower amount of sarcopenic people are found. Although previous research has shown promising results regarding SARC-CalF, with a better sensitivity than SARC-F [21,39], this does not concur with our results. This may be explained by the cut-offs that have been used, since again different options are found in this regard [37], and should be addressed in future research. Moreover, the few participants that were detected as suffering from sarcopenia with any of the options of Model 3 were classified as severe sarcopenia, thus indicating they were highly impaired in their physical performance, which allows less options of recovery. From the clinical approach, if a sarcopenia screening test is used, it is expected to dismiss from further testing as many healthy individuals as possible but should also guarantee diagnosis of those who do have sarcopenia [39] in order to start the appropriate intervention, thus this may not be possible using the SARC-CalF in a population like ours.

Considering that from the three models, Model 2 has shown the highest positive associative probability in case finding of participants with sarcopenia, especially in probable and confirmed, this finding would allow clinicians to detect sarcopenia in earlier stages. This model has different options which have shown statistical differences and using one or other to detect the presence of sarcopenia can be time consuming and expensive and might require highly specialized equipment [50]. Moreover, selecting a way of diagnosing sarcopenia requires balancing the possible benefit of including functional and ASM measurements against the difficulties related to their inclusion [62]. Therefore, on the whole, those options of Model 2 which include the chair stand and use the ASM may be finding more cases of sarcopenia in its different classifications.

### Limitations and Strengths

The main limitation of our study, common to other studies, is related to sample size. A larger sample size would be advisable, as well as studying case finding and implementing this model analysis in other populations besides the Spanish one to confirm our promising results. Another limitation is that the sample had a higher percentage of women, and although this is characteristic related to aged population in Spain, greater gender equality would be important in future research. Another interesting line to be implemented in the future could be analyzing how those older adults found to be sarcopenic in one model behave in the other models. However, this study offers the novelty of analyzing the different options of the EWGSOP2 to show the one that can find sarcopenia cases in an accurate way, which would promote an adequate and early intervention.

## 5. Conclusions

There are differences in case finding of sarcopenia in the studied Spanish older adults when the different measurement options for each step of the EWGSOP2 definition are applied. For muscle strength, the chair stand seems to be detecting more cases of probable sarcopenia, for muscle mass, ASM detects more confirmed and severe, and for physical performance, SPPB and gait speed seem to be reliable options. In addition, more sarcopenia cases are identified when no initial screening is used, therefore, in clinical practice, when a patient shows symptoms or signs of sarcopenia, a screening questionnaire may be surpassed and further testing is recommended to confirm sarcopenia. Thus, clinical settings should take into consideration that the methods used to define these steps can make preventive and therapeutic interventions on sarcopenia vary widely.

## Figures and Tables

**Table 1:** Description of the three models and their 12 options.

Name of Combination	Model *	Option	Muscle Strength Measurement	Muscle Quantity Measurement	Physical Performance Measurement
**A1**	1	A	Handgrip strength	ASMI	SPPB
**A2**	2				
**A3**	3				
**B1**	1	B	Handgrip strength	ASM	SPPB
**B2**	2				
**B3**	3				
**C1**	1	C	Handgrip strength	ASM	Gait speed
**C2**	2				
**C3**	3				
**D1**	1	D	Handgrip strength	ASMI	Gait speed
**D2**	2				
**D3**	3				
**E1**	1	E	Handgrip strength	ASM	TUG
**E2**	2				
**E3**	3				
**F1**	1	F	Handgrip strength	ASMI	TUG
**F2**	2				
**F3**	3				
**G1**	1	G	Chair stand	ASMI	SPPB
**G2**	2				
**G3**	3				
**H1**	1	H	Chair stand	ASM	SPPB
**H2**	2				
**H3**	3				
**I1**	1	I	Chair stand	ASM	Gait speed
**I2**	2				
**I3**	3				
**J1**	1	J	Chair stand	ASMI	Gait speed
**J2**	2				
**J3**	3				
**K1**	1	K	Chair stand	ASM	TUG
**K2**	2				
**K3**	3				
**L1**	1	L	Chair stand	ASMI	TUG
**L2**	2				
**L3**	3				

* Model 1: using SARC-F as initial screening; Model 2: not using any initial screening; and Model 3: using SARC-CalF instead of SARC-F.

**Table 2:** Characteristics of the participants (*n* = 272) according to setting and gender: mean (standard deviation) and [95% confidence interval].

	Community Dwelling (*n* = 139, 51.1%)				Institutionalized *(n* = 133, 48.9%)			
Variable	Total	Men (*n* = 44, 31.7%)	Women (*n* = 95, 68.3%)	*p*-value	Total	Men (*n* = 31, 23.3%)	Women (*n* = 102, 76.7%)	*p*-Value
**Anthropometrics**								
Age (years)	72.3 (6.1) [71.2–73.3]	72.8 (6.32) [70.9–74.7]	72.0 (6.1) [70.8–73.3]	0.498	81.9 (8.4) [80.5–83.3]	78.2 (9.0) [74.9–81.5]	83.0 (7.9) [81.5–84.6]	0.005 *
Weight (kg)	71.6 (12.4) [69.5–73.7]	79.5 (10.5) [76.3–82.7]	67.9 (11.5) [65.6–70.3]	<0.001 ^†^	66.6 (13.4) [64.4–69.0]	75.6 (12.5) [71.0–80.2]	63.9 (12.5) [61.5–66.4]	<0.001 ^†^
Height (cm)	158.9 (7.8) [157.6–160.2]	166.5 (6.8) [164.4–168.6]	155.4 (5.3) [154.3–156.5]	<0.001 ^†^	154.1 (9.1) [152.5–155.6]	164.9 (7.9) [162.0–167.8]	150.8 (6.5) [149.5–152.0]	<0.001 ^†^
BMI (kg/m^2^)	28.3 (4.2) [27.6–29.0]	28.7 (3.7) [27.5–29.8]	28.1 (4.4) [27.2–29.0]	0.486	28.0 (4.9) [27.2–28.9]	27.8 (3.8) [26.4–29.2]	28.1 (5.2) [27.1–29.1]	0.075
Calf circumference	36.3 (3.2) [35.7–36.8]	37.5 (3.1) [36.6–38.5]	35.7 (3.0) [35.1–36.3]	0.001 *	33.4 (3.4) [32.8–34.0]	33.9 (3.0) [32.8–35.0]	33.3 (3.5) [32.6–34.0]	0.387
**EWSGOP2 algorithm**								
SARC-F (0–10 score)	0.7 (1.2) [0.5–0.9]	0.3 (0.6) [0.1–0.5]	0.9 (1.3) [0.7–1.2]	<0.001 ^†^	3.9 (2.6) [3.5 – 4.4]	3.5 (2.8) [2.5–4.5]	4.0 (6.5) [3.5–4.5]	0.330
SARC-CalF (0–20 score)	0.9 (2.0) [0.6–1.3]	0.5 (1.6) [0.04–1.0]	1.1 (2.1) [0.7–1.6]	0.088	5.6 (5.1) [4.7–6.5]	4.8 (5.1) [2.9–6.7]	5.8 (5.2) [4.8–6.9]	0.338
Grip strength	28.4 (9.0) [26.9–29.9]	38.1 (8.4) [35.6–40.7]	23.8 (4.7) [22.9–24.8]	<0.001 ^†^	18.8 (7.8) [17.4–20.1]	26.6 (9.8) [23.0–30.2]	16.4 (5.1) [15.4–17.4]	<0.001 ^†^
Chair Stand	15.3 (13.0) [13.1–17.5]	11.4 (3.5) [10.4–12.5]	17.1 (15.2) [14.0–20.2]	0.001 *	31.2 (20.0) [27.8–34.7]	29.4 (19.2) [22.4–36.5]	31.8 (20.3) [27.8–35.8]	0.564
ASM (kg)	17.8 (4.0) [17.1–18.5]	22.3 (2.8) [21.4–23.2]	15.8 (2.5) [15.3–16.3]	<0.001 ^†^	15.1 (3.5) [14.5–15.7]	19.5 (3.2) [18.3–20.7]	13.8 (2.3) [13.4–14.3]	<0.001 ^†^
ASM/height^2^ (kg/m^2^)	7.0 (1.1) [6.8–7.2]	8.1 (0.9) [7.8–8.3]	6.5 (0.9) [6.3–6.7]	<0.001 ^†^	6.3 (1.0) [6.2–6.5]	7.2 (0.8) [6.8–7.5]	6.1 (0.9) [5.9–6.2]	<0.001 ^†^
Gait speed (m/s)	1.1 (0.3) [1.1–1.2]	1.2 (0.2) [1.1–1.3]	1.1 (0.3) [1.0–1.1]	0.012 *	0.6 (0.3) [0.5–0.6]	0.6 (0.3) [0.5–0.7]	0.6 (0.3) [0.5 –0.6]	0.576
SPPB (0–12 score)	10.4 (2.0) [10.1–10.8]	11.1 (1.1) [10.8–11.4]	10.4 (2.0) [9.6–10.6]	0.001 *	5.3 (3.0) [4.8 –5.8]	6.1 (2.8) [5.1–7.2]	5.0 (3.0) [4.4–5.6]	0.064
TUG (s)	9.8 (3.3) [9.2–10.3]	9.1 (2.0) [8.5–9.7]	10.1 (3.7) [9.3–10.8]	0.044 *	26.9 (18.7) [23.6–30.1]	28.8 (24.1) [20.0–37.6]	26.3 (16.7) [23.0–29.6]	0.588

Abbreviations: BMI = Body Mass Index; ASM = Appendicular Skeletal Muscle Mass; SPPB = Short Physical Performance Battery; TUG = Timed-Up and Go test. *p*-value unpaired Student’s t-test. * *p* < 0.05; ^†^ *p* < 0.001.

**Table 3:** Goodness-of-fit chi-square tests, probability level corrected with Sidak method.

Model 1	χ^2^	df	*p*-Value	Model 2	χ^2^	df	*p*-Value	Model 3	χ^2^	df	*p*-Value
A1	10.45	3	>0.05	A2	30.43	3	<0.05	A3	1.64	3	>0.05
B1	21.47	3	<0.05	B2	48.49	3	<0.05	B3	3.12	3	>0.05
C1	21.47	3	<0.05	C2	44.05	3	<0.05	C3	3.12	3	>0.05
D1	10.45	3	>0.05	D2	27.92	3	<0.05	D3	1.64	3	>0.05
E1	31.86	3	<0.05	E2	57.01	3	<0.05	E3	8.39	3	>0.05
F1	10.45	3	<0.05	F2	34.16	3	<0.05	F3	8.12	3	>0.05
G1	37.98	3	<0.05	G2	72.80	3	<0.05	G3	4.35	3	>0.05
H1	26.07	3	<0.05	H2	56.71	3	<0.05	H3	5.78	3	>0.05
I1	22.79	3	<0.05	I2	46.70	3	<0.05	I3	5.78	3	>0.05
J1	37.98	3	<0.05	J2	69.28	3	<0.05	J3	4.35	3	>0.05
K1	41.36	3	<0.05	K2	90.20	3	<0.05	K3	14.45	3	<0.05
L1	42.65	3	<0.05	L2	81.24	3	<0.05	L3	9.56	3	>0.05

Notes: Model 1: using SARC-F; Model 2: not using any initial screening; Model 3: using SARC-CalF; *p*-values corrected with Sidak’s correction.
